# Cisplatin Protects against Acute Liver Failure by Inhibiting Nuclear HMGB1 Release

**DOI:** 10.3390/ijms140611224

**Published:** 2013-05-27

**Authors:** Xun Li, Li-Kun Wang, Lu-Wen Wang, Xiao-Qun Han, Fan Yang, Zuo-Jiong Gong

**Affiliations:** Department of Infectious Diseases, Renmin Hospital of Wuhan University, Wuhan University, Wuhan 430060, China; E-Mails: xunlee2011@gmail.com (X.L.); lkwang999@163.com (L.-K.W.); wangluw8253@163.com (L.-W.W.); hxqqqqq520@126.com (X.-Q.H.); yangfanwhu2012@163.com (F.Y.)

**Keywords:** cisplatin, acute liver failure, high mobility group box-1, HMGB1 translocation, inflammation

## Abstract

Cisplatin is one of the most widely used chemical drugs for anticancer treatment. Recent studies have focused on the ability of cisplatin to retain the high mobility group box 1 (HMGB1) protein in cisplatin-DNA adducts, thereby preventing its release from the nucleus. Because HMGB1 is a powerful inflammatory mediator in many diseases, the aim of this study is to evaluate the therapeutic effect of cisplatin acute liver failure. In this study, low-dose cisplatin was administered to treat PMA-induced macrophage-like cells induced by PMA and rats with acute liver failure. We found that cell viability and liver injury were greatly improved by cisplatin treatment. The extracellular levels of HMGB1, TNF-α and IFN-γ were also significantly decreased by the administration of cisplatin. During inflammation, nuclear HMGB1 translocates from the nucleus to the cytoplasm. The administration of cisplatin reduced the cytoplasmic levels of HMGB1 and increased nuclear HMGB1 levels *in vitro* and *in vivo*. In conclusion, cisplatin can protect against acute liver failure by retaining HMGB1 in the nucleus and preventing its release into the extracellular milieu.

## 1. Introduction

High mobility group box 1 (HMGB1) is a non-histone DNA-binding protein that has been identified as a chromatin architectural factor that facilitates gene transcription, DNA replication and repair by mediating DNA bending [1–3]. In addition to its intranuclear function, the extracellular function of HMGB1 has also been studied in detail. Many studies have shown that extracellular HMGB1 functions as a cytokine. As an inflammatory mediator, HMGB1 plays a crucial role in many diseases including acute pancreatitis [4], acute lung injury [5], rheumatoid arthritis [6], hemorrhagic shock [7] and ischemia-reperfusion injury [8]. Treatment with anti-HMGB1 agents, either chemical or biological, could alleviate inflammatory responses in these diseases. Our previous study (in press) demonstrated that treatment with an anti-HMGB1 antibody protects against acute-on-chronic liver failure in rats.

Cisplatin is one of the most widely used anticancer chemical drugs. It has been used to treat a range of tumors including head and neck, lung, breast, ovarian and cervical cancers [9]. As an anticancer agent, cisplatin exerts its cytotoxic role in tumor cells by damaging DNA through the formation of covalent bonds with purine bases [10]. Fundamental cellular processes, including replication, transcription, translation and DNA repair are inhibited by the covalent DNA–platinum adducts [11]. It has been reported that cisplatin treatment at relatively high doses results in the production of significant amounts of nitric oxide (NO) and pro-inflammatory cytokines in macrophages and exhibits increased tumoricidal activity [12]. Moreover, low-dose cisplatin administration in murine cecal ligation and puncture (CLP) has been shown to prevent the systemic release of HMGB1 and attenuate lethality [13].

HMGB1 binds to platinized lesions on DNA with a specificity for 1, 2-intrastrand cross-links, which account for the cisplatin–DNA adducts formed mostly *in vivo* [14]. Considering the ability of cisplatin to sequester HMGB1 and the cytokine activity of HMGB1, it is reasonable to hypothesize that cisplatin could be used for the treatment of inflammation. Nontoxic low-dose cisplatin treatment studies have also shown that cisplatin can confer protection against sepsis and ischemia/reperfusion injury in mice [13,15]. To explore whether cisplatin can be used as a new drug for acute liver failure (ALF) treatment, we evaluated the effects of cisplatin on U937 cells and in the ALF model in rats induced by D-Gal combined with LPS.

## 2. Results and Discussion

### 2.1. Effect of Cisplatin on the Viability of Macrophage-Like Cells

**Figure 1:**
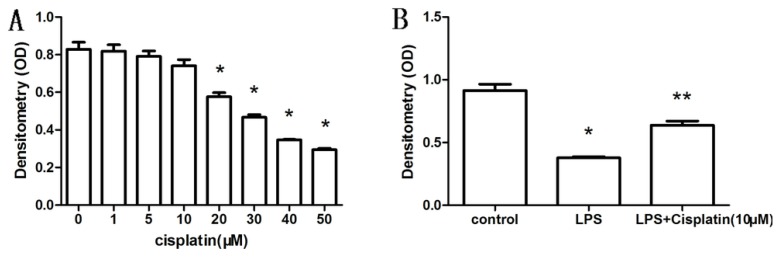
Viability of macrophage-like cells measured by CCK-8 assay. (**A**) Toxic effect of cisplatin (0–50 μM), relative to medium without cisplatin, *p < 0.05; (**B**) Protective effect of low-dose cisplatin. Cell viability is presented as the densitometry value. A higher densitometryvalue indicates higher viability of macrophage-like cells. Compared to the control group, *p < 0.05; compared to the LPS-treated group, **p < 0.05. Results are expressed as the mean ± SD (*n* = 4).

The viability of macrophage-like cells was measured by CCK-8 assay. Cell viability was not decreased by cisplatin at concentrations below 10 μM (Figure 1A). However, viability of the cells decreased remarkably when LPS (1 μg/mL) was added to the culture medium (*p* < 0.05), and improved when 10 μM cisplatin was added (Figure 1B).

### 2.2. Effect of Cisplatin on Cytokine Production in Macrophage-Like Cells

**Figure 2:**
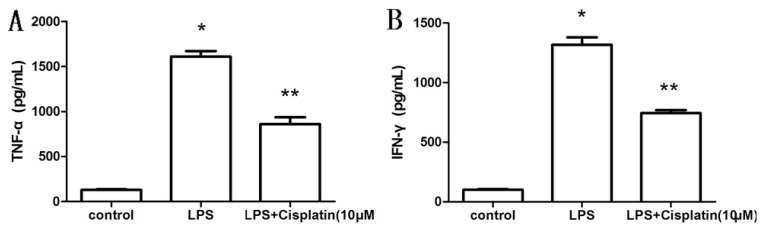
Effect of cisplatin on TNF-α and IFN-γ in macrophage-like cells. (**A**) TNF-α level; (**B**) IFN-γ level. Compared with the control group, *p < 0.05; compared with the LPS-treated group, **p < 0.05. Results are expressed as the mean ± SD (*n* = 4).

Previous data has led us to believe that a low dose of cisplatin can modulate immunoresponses. To test this hypothesis, we measured TNF-α and IFN-γ in cell supernatants after LPS exposure with and without low dose Cisplatin. The levels of TNF-α and IFN-γ in cell supernatants were measured |by ELISA.TNF-α and IFN-γ levels were significantly increased in LPS-treated group (1611.25 ± 123.24 pg/mL and 1317.50 ± 124.48 pg/mL, respectively) relative to the control group (131.5 ± 7.72 pg/mL and 100.25 ± 11.17 pg/mL, respectively) (*p* < 0.05). However, TNF-α and IFN-γ levels were markedly reduced by 46.54% and 43.47% in the LPS + cisplatin-treated group (861.25 ± 149.50 pg/mL and 744.75 ± 48.77 pg/mL, respectively) (*p* < 0.05) (Figure 2). These data show that cisplatin somehow reduce the sera expression of the inflammatory mediators.

### 2.3. Effect of Cisplatin on HMGB1 in Macrophage-Like Cells

**Figure 3:**
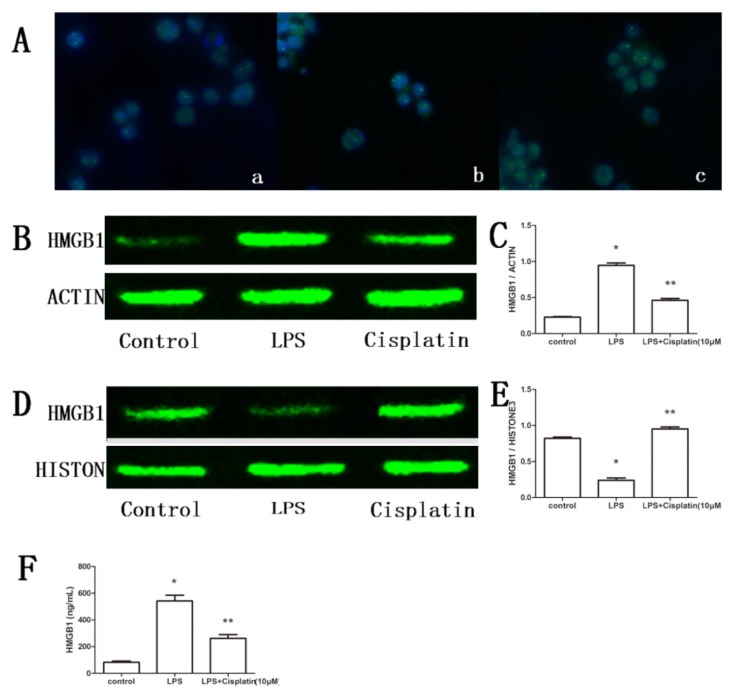
Effect of cisplatin on HMGB1in macrophage-like cells. (**A**) Immunofluorescence analysis of HMGB1. HMGB1 was stained green, and nuclei were stained blue, (original magnification ×400). a: Control group, HMGB1 (green fluorescence) was localized mainly in the nucleus; b: LPS-treated group, HMGB1 was localized mainly in the cytoplasm and extracellular matrix; c: LPS+cisplatin-treated group, although HMGB1 present both in nucleus and extracellular matrix, HMGB1 mainly existed in the nucleus; (**B**) Cytoplasmic levels of HMGB1 detected by Western blot analysis. β-actin was probed as an additional loading control. Control: control group, LPS: LPS-treated group, CISPLATIN: LPS + cisplatin-treated group; (**C**) Semi-quantification of cytoplasmic levels of HMGB1. The relative protein expression levels from three independent experiments are represented as the density of HMGB1 bands normalized to that of β-actin bands. Compared with the control group, *p < 0.05; compared with the LPS-treated group, **p < 0.05. Results are expressed as the mean ± SD (*n* = 3); (**D**) Nuclear levels of HMGB1 detected by Western blot analysis. Histone3 was probed as an additional loading control. Control: control group, LPS: LPS-treated group, CISPLATIN: LPS + cisplatin-treated group; (**E**) Semi-quantification of nuclear levels of HMGB1. The relative protein expression levels from three independent experiments are represented as the density of HMGB1 bands normalized to that of Histone3 bands. Compared with the control group, *p< 0.05; compared with the LPS-treated group, **p< 0.05. Results are expressed as the mean ± SD (*n* = 3); (**F**) The supernatant levels of HMGB1. Compared to the control group, *p< 0.05; compared with the LPS-treated group, **p< 0.05. Results are expressed as the mean ± SD (*n* = 4).

To observe the changes of HMGB1 in location, immunofluorescence experiments were performed. The results showed that HMGB1 exists mainly in the nucleus of macrophage-like cells. LPS treatment induces translocation of HMGB1 from the nucleus to the cytoplasm and extracellular matrix. In the LPS + cisplatin-treated group of macrophage-like cells, HMGB1 was retained in the nucleus (Figure 3A). To confirm the changes of HMGB1 observed in immunofluorescence, HMGB1 levels in macrophage-like cells and cell supernatants were determined by Western blot analysis and ELISA, respectively. The ELISA results showed that supernatantHMGB1 levels increased greatly in the LPS-treated group (542.42 ± 86.41 ng/mL) relative to the control group (82.94 ± 18.16 ng/mL) (*p* < 0.05). However, in the LPS + cisplatin-treated group, supernatant HMGB1 levels (262.65 ± 55.30 ng/mL) decreased by 51.58% relative to the LPS-treated group (*p* < 0.05) (Figure 3F). Western blot analysis of macrophage-like cells showed that the cytoplasmic levels of HMGB1 increased evidently in the LPS-treated group (0.946 ± 0.060) relative to the control group (0.227 ± 0.014) (*p* < 0.05), whereas the relative levels of HMGB1 were reduced by 51.27% in the LPS+cisplatin-treated group (0.461 ± 0.046) relative to the LPS-treated group (*p* < 0.05) (Figure 3B,C). In addition, nuclear HMGB1 levels decreased in the LPS-treated group (0.238 ± 0.057) relative to the control group (0.822 ± 0.030) (*p* < 0.05), whereas relative levels of HMGB1 increased by 299.58% in LPS + cisplatin-treated group (0.951 ± 0.051) relative to the LPS-treated group(*p* < 0.05) (Figure 3D,E). These data indicate that cisplatin can prevent the translocation of HMGB1 from the nucleus to the cytoplasm and decrease extracellular HMGB1 level.

### 2.4. Effect of Cisplatin on ALT Level and Apoptosis of Hepatic Tissue

**Figure 4:**
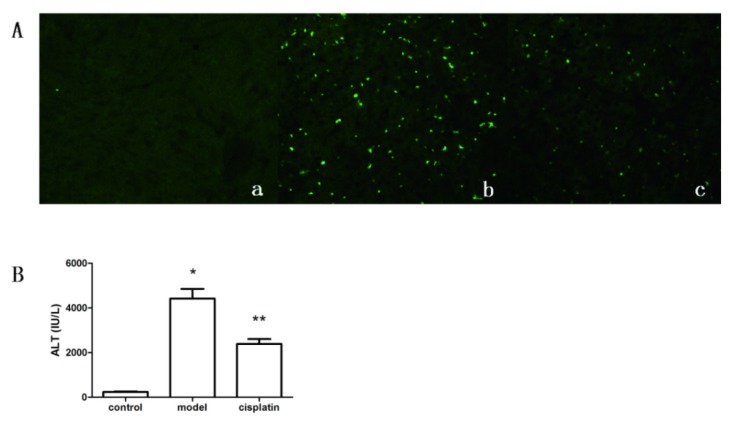
Effect of cisplatin on serum ALT levels and apoptosis in hepatic tissue. (**A**) Apoptosis in hepatic tissue was detected by TUNEL assay, where apoptosis cells were stained green. a: Control group, apoptosis cells were rarely observed; b: Model group, many apoptosis cells were observed; c: Cisplatin treatment group, relatively fewer apoptosis cells were observed relative to the model group; (**B**) ALT levels in serum, control: Control group, model: Model group, cisplatin: Cisplatin treatment group. Compared to the control group, *p < 0.05; compared to the model group, **p < 0.05. Results are expressed as the mean ± SD (*n* = 5).

Because cisplatin exerts its anti-tumor effect through the activation of apoptosis, the apoptosis of hepatic tissue was measured to evaluate the apoptosis of hepatocytes when ALF occurred. TUNEL assay showed that apoptosis was rarely observed in the control group, and was evidently increased after ALF induction in the model group. However, compared with the model group, less apoptosis was observed in the cisplatin treatment group (Figure 4A). To determine whether the model was induced successfully and the protective effect of cisplatin on ALF, ALT levels in sera were detected. The results (Figure 4B) showed that ALT levels increased markedly in the model group (4418.87 ± 86.92 IU/L) compared to the control group (136.60 ± 41.96 IU/L) (*p* < 0.05), and cisplatin administration attenuated ALT levels by 45.82% in the cisplatin treatment group (2394.01 ± 430.62 IU/L) (*p* < 0.05). These results suggest that the ALF model is induced successfully with D-Gal and LPS, and treatment with a low dose of cisplatin reduces apoptosis and necrosis of liver cells in ALF.

### 2.5. Effect of Cisplatin on Survival Time

**Figure 5:**
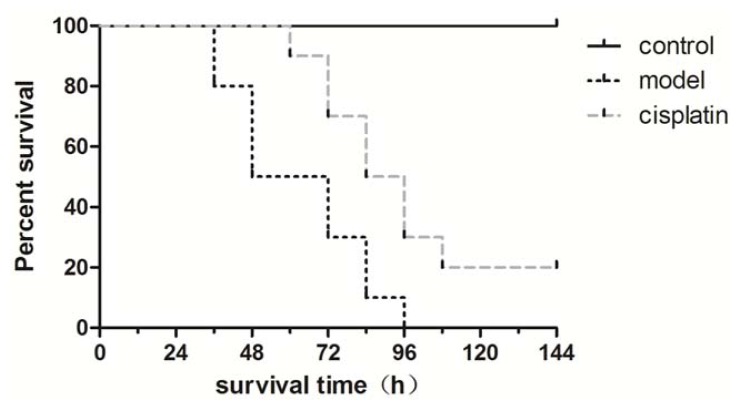
Effect of cisplatin on the survival time of rats.

To assess the outcome of cisplatin in treating ALF, the survival time of rats were observed. All of the ALF rats died within four days without cisplatin treatment, and their median survival time was 60 h. However, cisplatin treatment prolonged the median survival time of the ALF rats to 90 h. As determined by log-rank test, there was a significant difference between the survival curves for the two groups (*χ*^2^ = 22.21, *p* < 0.0001) (Figure 5). The result indicates that low-dose cisplatin administration increases survival rate of rats in ALF.

### 2.6. Effect of Cisplatin on Cytokine Production in ALF

**Figure 6:**
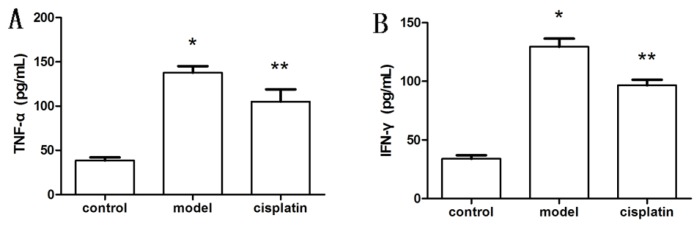
Effect of cisplatin on TNF-α and IFN-γ in ALF. (**A**) Serum levels of TNF-α; (**B**) Serum levels of IFN-γ. Control: Control group, model: Model group, cisplatin: Cisplatin treatment group. Compared to the control group, *p < 0.05; compared to the model group, **p < 0.05. Results are expressed as the mean ± SD (*n* = 5).

To evaluate whether the effect of cisplatin on cytokine production was consistent with that in inflammatory cells, TNF-α and IFN-γ levels were also detected in rats with ALF. As shown in Figure 6, TNF-α and IFN-γ levels increased remarkably in the model group (137.73 ± 7.35 pg/mL and 129.46 ± 6.99 pg/mL, respectively) relative to the control group (38.65 ± 3.37 pg/mL and 33.90 ± 3.09 pg/mL, respectively). The levels of these cytokines decreased by 23.72% and 25.41% after administration of cisplatin in the model group (105.06 ± 13.89 pg/mL and 96.57 ± 4.69 pg/mL, respectively) (*p* < 0.05). The data suggest that as demonstrated *in vitro*, a low dose of cisplatin is also able to reduce cytokine production *in vivo*.

### 2.7. Effect of Cisplatin on HMGB1 in ALF

**Figure 7:**
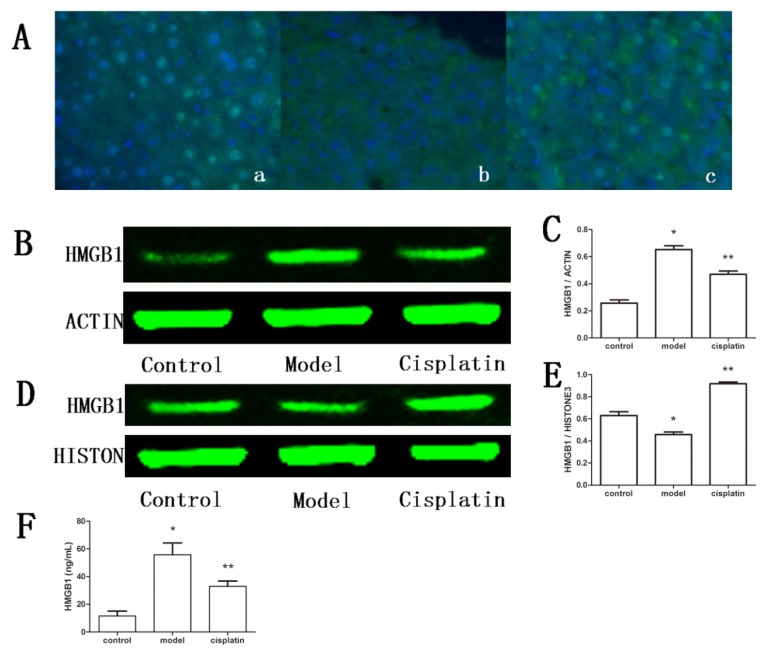
Effect of cisplatin on HMGB1inALF. (**A**) Immunofluorescence staining of HMGB1 in hepatic tissue. HMGB1 was stained green, and nuclei were stained blue, (original magnification ×400). a: Control, HMGB1 (green fluorescence) was observed mainly in nucleus; b: Model group, HMGB1 was observed mainly in the cytoplasm; c: Cisplatin treatment group, HMGB1 was found in the nucleus and cytoplasm; (**B**) Cytoplasmic levels of HMGB1 detected by Western blot analysis. β-actin was probed as an additional loading control. Control: Control group, model: Model group, Cisplatin: Cisplatin treatment group; (**C**) Semi-quantification of cytoplasmic HMGB1 levels. The values for expression are from three independent experiments and are represented as the density of HMGB1 bands normalized to that of β-actin bands. Compared to the control group, *p < 0.05; compared to the model group, **p < 0.05. Results are expressed as the mean ± SD (*n* = 3); (**D**) Nuclear levels of HMGB1 detected by Western blot analysis. Histone3 was probed as an additional loading control. Control: Control group, Model: Model group, Cisplatin: Cisplatin treatment group; (**E**) Semi-quantification of nuclear HMGB1 levels. The values of relative protein expression levels are from three independent experiments and are represented as the density of HMGB1bands normalized to Histone3 bands. Compared to the control group, *p < 0.05; compared to the model group, **p < 0.05. Results are expressed as the mean ± SD (*n* = 3); (**F**) Serum levels of HMGB1. Control: Control group, model: Model group, cisplatin: Cisplatin treatment group. Results are expressed as the mean ± SD (*n* = 5).

Similar to the results seen in macrophage-like cells, the results of immunofluorescence of hepatic tissue showed that HMGB1 existed mainly in the nucleus of liver cells in the control group. However, upon induced ALF, HMGB1 translocated from the nucleus to cytoplasm, whereas HMGB1 was retained in the nucleus in the cisplatin treatment group (Figure 7A). HMGB1 levels in rat sera and hepatic tissue lysates were determined by ELISA and Western blot analysis, respectively. The ELISA results showed that the serum levels of HMGB1 increased greatly in the model group (55.77 ± 8.46 ng/mL) relative to the control group (11.57 ± 3.53 ng/mL) (*p* < 0.05) but decreased by 40.90% in the cisplatin treatment group (32.96 ± 3.81 ng/mL) relative to the model group (*p* < 0.05) (Figure 7F). Western blot analysis of HMGB1 levels in hepatic tissue showed that cytoplasmic levels of HMGB1 increased in the model group (0.652 ± 0.049) relative to the control group (0.257 ± 0.041), but relative nuclear HMGB1 levels in the model group (0.458 ± 0.039) were markedly lower than those of the control group (0.629 ± 0.061) (*p* < 0.05). Treatment with cisplatin reduced the cytoplasmic HMGB1 protein levels by 25% in hepatic tissue (0.470 ± 0.041), and increased the nuclear HMGB1 levels by 100.48% (0.918 ± 0.028) (*p* < 0.05) (Figure 7B–E). The data demonstrate that a low dose of cisplatin is able to inhibit HMGB1 translocation in ALF.

ALF is a severe syndrome with high lethality, and its pathological mechanisms are not yet fully understood. Many factors have been implicated in the pathogenesis of the disease including endotoxin and inflammatory cytokines [16–18]. Intraperitoneal injection with D-Gal and LPS to induce acute liver injury is a classic model for ALF research. The monocyte-like cell line U-937 is widely used in inflammation research, when differentiated by phorbol myristate acetate (PMA) and activated by lipopolysaccharide (LPS) [19]. Because inflammatory cells play an important role in the pathogenesis of ALF, we utilized differentiated U937 cells and an acute liver failure model using D-Gal and LPS to evaluate the therapeutic effects of cisplatin on ALF.

Theoretically, cisplatin exerts its biological effects by interacting with DNA to form DNA adducts, which activate several signal transduction pathways, including those involving ATR, p53, p73 and MAPK, thereby culminating in the activation of apoptosis [20]. Many proteins such as mismatch repair (MMR) complex, high-mobility groups 1 and 2 (HMG1 and HMG2) proteins, upstream binding factor (hUBF), and TATA binding protein (TBP) contribute to these pathway by binding to the cisplatin–DNA adducts [20]. Since its first demonstrated in ischemia/reperfusion injury [15], the anti-inflammation role of cisplatin has sequentially been confirmed in CLP-induced sepsis [13] and post-transplantation pancreatitis [21]. In this study, we demonstrate that a nontoxic dose of cisplatin can improve the viability of macrophage-like cells and significantly attenuated liver cells injury and apoptosis. The survival time of the animals was also prolonged by cisplatin treatment. These results indicate that a nontoxic dose of cisplatin is beneficial for ALF.HMGB1 has been identified as a secreted extracellular mediator of inflammation and repair responses in LPS-induced endotoxemia and sepsis [22]. It is actively secreted by immunostimulated macrophages [23–26] and enterocytes [27] and is also released by necrotic, but not apoptotic cells [28]. Once released into the extracellular milieu, HMGB1 binds to many extracellular receptors, such as RAGE, TLR2, TLR4 and TLR9 [29–31]. By interacting with different receptors, HMGB1 mediates a variety of responses including epithelial barrier dysfunction, the release of pro-inflammatory cytokines and acute inflammation [32]. In addition, the administration of HMGB1 to normal animals leads to systemic inflammatory responses [29], which suggest a role for extracellular HMGB1 in the induction of inflammatory responses. In this study, we also measured the levels of TNF-α and IFN-γ in the supernatant of macrophage-like cells and serum from rats, our results show that TNF-α and IFN-γ levels were also decreased by the administration of cisplatin *in vitro* and *in vivo*.

In this study, we showed that the production of inflammatory cytokines is attenuated by cisplatin. To determine whether this effect was associated with extracellular HMGB1, the levels of HMGB1 in the serum and cell supernatant were measured. Extracellular levels of HMGB1 increased significantly both *in vitro* and *in vivo*, and this increase was attenuated by cisplatin treatment. Cisplatin can bind to DNA and cause a dramatic distortion in the duplex structure. HMGB1 is able to recognize the distorted DNA structures and binds to them stably, especially via the formation of 1, 2-intrastrand cross-links [33]. The main effect of cisplatin on HMGB1 known so far is that it sequesters HMGB1 to the cisplatin-DNA adducts and prevents its release from the nucleus to the cytoplasm and extracellular milieu, this effect of cisplatin on HMGB1 in macrophage-like cells and hepatic tissue was evaluated by immunofluorescence. As demonstrated in this study, HMGB1 exists mainly in the nucleus of macrophage-like cells and liver cells, and the translocation of HMGB1 occurs when cells are stimulated by LPS or after ALF induction. To determine whether cisplatin treatment could suppress the release of HMGB1 from the nucleus to the extracellular milieu, HMGB1 levels in macrophage-like cells and hepatic tissue were also determined. There were higher levels of HMGB1 in the nucleus and lower levels were observed in the cytoplasm in the cisplatin-treated group relative to the model group.

Although the cause and mechanism of ALF pathogenesis are still unclear, liver cell necrosis and inflammatory cell infiltration can be observed in disease-affected tissue. In this study, the effects of cisplatin were evaluated both *in vitro* and *in vivo*, and hepato-protective effect of cisplatin was demonstrated in rats with ALF. Our results show that extracellular HMGB1 plays a key role in the inflammatory responses during ALF pathogenesis and that cisplatin reduces extracellular HMGB1 levels by inhibiting the translocation of HMGB1 from the nucleus to the cytoplasm and extracellular milieu.

## 3. Experimental Section

### 3.1. Cell Culture and Treatment

U-937 cells (ATCC, Manassas, VA, USA) were cultured and maintained at a cell density between 2 × 10^6^ and 1 × 10^7^ cells/mL in RPMI 1640 (Jinuo, Hangzhou, China) supplemented with 10% fetal bovine serum (Sijiqing, Huzhou, China), 100 U/mL penicillin, and 100 μg/mL streptomycin. For macrophage-like cell differentiation, serum starved U-937 cells were plated in a 35-mm cell culture dish (2 × 10^6^ cells/mL) and treated with 60 ng/mL PMA for 24 h. The non-adherent cells were removed by two PBS washes. Complete RPMI 1640 medium containing 1 μg/mL LPS or 1 μg/mL LPS combined with cisplatin (different doses) was applied in subsequent experiments. Cultural supernatants were collected for cytokine detection.

### 3.2. Animals

Male-specific pathogen-free (SPF) Wistar rats, weighing 180 to 220 g, were purchased from the Experimental Animal Center of Wuhan University. All animals were housed in a light-controlled room (12 h light/dark cycle) at an ambient temperature of 25 °C with free access to water and standard chow. All animals were given humane care in accordance with institutional guidelines.

### 3.3. Model Production and Sample Collection

The animals were randomly divided into three groups: normal group, model group and cisplatin treatment group. The rat ALF model was induced by D-Gal and LPS. The rats in the model group (*n* = 15) received 400 mg/kg D-Gal 100 μg/kg LPS by intraperitoneal injection. In addition to D-Gal and LPS, the rats in the cisplatin treatment group (*n* = 15) received 100 μg/kg cisplatin [14] by intraperitoneal injection 2 h after ALF induction. The rats in the control group (*n* = 15) were injected intraperitoneally with normal saline solution (NSS) as a control. The time of administration of D-Gal combined with LPS was assigned as the baseline (time point 0). Five animals from each group were sacrificed for blood and hepatic tissue collection at the 24 h time point. The remaining 10 rats were observed for survival time every 12 h for 7 days after induction of the ALF.

### 3.4. Cell Viability Test

Cell viability was assessed using Cell Counting Kit-8 (Dojindo, Shanghai, China) assay. Cells (2 × 10^5^ cells/well) were plated in 96-well plates for viability measurement. Experimental procedures were performed according to the manufacturer’s protocol.

### 3.5. TUNEL Assay

Portions of liver samples were fixed in 10% buffered formalin for 24 h, embedded in paraffin and sliced into sections of 5-μm thickness. The sections were incubated in 0.1% Triton X-100 for 8 min and washed twice in PBS for 5 min each. *In situ* labeling of apoptosis-induced DNA strand breaks (TUNEL assay) was performed using a commercially available kit (*in situ* Cell Death Detection Kit, Roche, Switzerland).

### 3.6. Analysis of Cell Supernatants and Blood Samples

The levels of HMGB1, TNF-α and IFN-γ in cell supernatantsand sera were measured using ELISA kits (HMGB1 from Westang, Shanghai, China, TNF-α and IFN-γ from Ebioscience, San Diego, CA, USA) according to the manufacturer’s recommendations. The alanine transaminase (ALT) level was determined by routine biochemical methods using a Hitachi Automatic Analyzer (Hitachi, Inc., Tokyo, Japan).

### 3.7. Extraction of Nuclear and Cytoplasmic Protein from Cells and Hepatic Tissue

Nuclear and cytoplasmic proteins were isolated by using a nuclear and cytoplasmic protein extraction kit (Beyotime, Nantong, China). Experimental procedures were conducted according to the manufacturer’s instructions.

### 3.8. Western Blot Analysis of HMGB1 Levels from U937 Cells and Hepatic Tissue

Briefly, 50 μg of different protein extracts was subjected to 10% SDS-PAGE and transferred onto a PVDF membrane (Millipore, Billerica, MA, USA). The membrane was incubated with anti-HMGB1 antibody (Epitomics, Burlingame, CA, USA), followed by incubation with a secondary antibody (LICOR, Lincoln, NE, USA), and then it was analyzed by ODESSEY infrared imaging system (LICOR, Lincoln, NE, USA). Membranes were also probed for beta-actin and histone 3 (Santa Cruz, CA, USA) as additional loading controls.

### 3.9. Immunohistological Analysis of HMGB1

Tissue sections were dewaxed in xylene and rehydrated in a series of dilutions of alcohol. The sections and cover glasses with fixed U937 cells were incubated in 0.1% Triton X-100 for 10 min, and washed twice in PBS for 5 min. The tissue sections were then placed in 3% H_2_O_2_ for 20 min to block endogenous peroxidase activity and boiled in sodium citrate buffer (pH = 6.0) for 15 min. The sections and fixed cells were blocked with 10% normal goat serum in a humid chamber at 37 °C for 20 min to reduce non-specific binding, and then incubated with a primary rabbit anti-HMGB1 antibody at a 1:500 dilution at 4 °C overnight. The sections were then incubated with fluorescein isothiocyanate (FITC)-conjugated goat anti-rabbit IgG (Boster Biotechnology, Wuhan, China) for 60 min at 37 °C in the dark, counterstained with 2 μg/mL 2-(4-Amidinophenyl)-6-indolecarbamidine dihydrochloride (DAPI) (Beyotime, Nantong, China) for 2 min and observed under animmunofluorescence microscope.

### 3.10. Statistical Analysis

Statistical analysis was performed using SPSS software (version 13.0 for Windows). Data are presented as the mean ± SD, and comparisons were made using Student’s *t* test and one-way ANOVA. Animal survival data were evaluated using the Kaplan–Meier method and compared using the log-rank test. A probability value of 0.05 or less was considered statistically significant.

## 4. Conclusions

Cisplatin confers protection against acute liver failure. The mechanism of protection potentially involves cisplatin-mediated sequestration of HMGB1 in the nucleus and inhibition of its translocation from the nucleus into the extracellular milieu.
